# ID 192 - Os efeitos da Suplementação Dietética para Crianças e Adolescentes com Doença Falciforme: uma revisão sistemática e metanálise

**DOI:** 10.5327/2237-9622.2025.v34s1.192

**Published:** 2025-11-25

**Authors:** Bruna Cardoso Orsi, Daniela Gorski, Naila Emilia Krul, Fernanda Stumpf Tonin, Roberto Pontarolo

**Keywords:** doença falciforme, suplementos dietéticos, crianças, adolescentes, revisão sistemática, metanálise

## Abstract

**Introdução:**

Doença falciforme (DF) é um distúrbio sanguíneo genético e negligenciado que impacta profundamente crianças e adolescentes e é associada a um comprometimento importante do estado nutricional. No Brasil, a taxa de mortalidade (doença falciforme como causa primária) é de 0,25 por 100 mil habitantes e, entre crianças entre 1 a 9 anos de idade, o risco de morte foi considerado 32 vezes maior quando comparado à população saudável. Este estudo tem como objetivo avaliar os efeitos de suplementos dietéticos como tratamento adjuvante nas complicações associadas à DF, bem como contribuir na atualização da evidência e das diretrizes sobre essa doença.

**Método:**

Uma revisão sistemática com busca nas bases PubMed, Scopus e Web of Science foi realizada (PROSPERO:CRD42024532369). Ensaios clínicos randomizados (ECR) que avaliaram suplementos dietéticos ou alimentos como tratamento adjuvante para crianças e adolescentes com DF foram incluídos. Desfechos de eficácia e de segurança foram coletados e sintetizados em uma metanálise em rede, com análise de ranking (p-score). Os resultados foram apresentados como razão de chances (OR) ou diferença média padronizada (SMD), para os dados dicotômicos e contínuos, respectivamente, com intervalos de confiança (IC) de 95% (NMAstudio2.0).

**Resultados:**

Vinte ECR foram incluídos (2002-2023) (n=2.058), avaliando nove intervenções em diferentes regimes terapêuticos. Todos os pacientes utilizavam hidroxiureia como tratamento ativo. A suplementação com ácidos graxos (n=3 estudos) e L-arginina (n=4) apresentaram maior eficácia e segurança, com melhora significativa na intensidade da dor, crises vaso-oclusivas (CVO) e inflamação, quando comparados ao placebo/tratamentos usuais (p<0,05). Estudos com vitamina D3 (n=6) utilizaram diferentes doses e foi observada uma redução em complicações respiratórias e tempo de hospitalização. As evidências sobre o uso complementar da vitamina A (n=2), sulfato de magnésio (n=2) e zinco (n=4) ainda são limitadas e de baixa qualidade nesta população.

**Conclusão:**

Neste estudo, o uso de alguns suplementos (ácidos graxos, L-arginina e vitamina D3) mostrou-se capaz de melhorar o manejo de CVO e funções fisiológicas destes pacientes. Já suplementos com vitamina A, sulfato de magnésio e zinco apresentam potenciais benefícios, porém, mais estudos são necessários para a confirmação destes efeitos. Suplementos dietéticos são comumente acessíveis e de baixo custo, além de contribuírem para a redução de uso de opioides e tempo de hospitalização – especialmente em países de baixa/média renda, onde recursos são escassos. Estudos realizados no Brasil mostram que episódios de dor representam a principal causa de hospitalização nesta população e CVO é a complicação mais prevalente em crianças (59,5%) com um importante impacto econômico: 87% dos custos de complicações são relacionados ao manejo de CVO em adultos. Ainda assim, o (2018) não menciona impactos clínicos de suplementos dietéticos como tratamento adjuvante (com exceção da suplementação com ácido fólico)(Brasil, 2018). Embora haja necessidade de novos estudos com dosagens e regimes apropriados para refinar a evidência, este estudo pode colaborar na tomada de decisões e diretrizes terapêuticas sobre DF na população pediátrica.

